# Therapeutic Notes

**Published:** 1895-02-16

**Authors:** 


### THERAPEUTIC NOTES.

### POISONING BY GUAIACOL.

Poisoning by guaiacol is so rare an event that little attention has been drawn to its possibility, but a fatal case has been reported by Professor Wyss, of Zurich (Deut. Med. Wochenschr., Mar. 28, 1894), which may teach us caution in its use. A girl, nine years old, was given inadvertently 75 minims guaiacol by the mouth. The symptoms which ensued were not unlike those of poisoning by carbolic acid, a body to which guaiacol is chemically closely allied. In a very short time there occurred unconsciousness, vomiting, rapid pulse and diminished sensibility of the skin, with lividity and frequent respiration. The stomach was washed out early, but in spite of this the symptoms did not improve; the collapse increased, hæmorrhages were observed under the skin, albuminuria and jaundice supervened, and the patient died on the third day. The guaiacol was partly excreted in the urine. At the autopsy acute gastro-enteritis, acute nephritis with hæmorrhages, ecchymoses into serous membranes, and degeneration of the liver substance and heart muscle were made out, with great enlargement of the spleen. Fifteen minims of guaiacol are known to produce slight degrees of poisoning, but little more than gastric pain with nausea and vomiting.

